# Isorhamnetin attenuates renal interstitial fibrosis by targeting TWEAK/Fn14-mediated epithelial–mesenchymal transition

**DOI:** 10.3389/fimmu.2025.1649327

**Published:** 2025-09-04

**Authors:** Yaping Chen, Wenchuan Luo, Hongxiang Guan, Zixin Chen, Zhihui Chen, Lijuan Xiao, Wen Xu, Mei Huang, Ya Lin, Yuqin Zhang, Weihua Peng, Lihong Nan

**Affiliations:** ^1^ College of Pharmacy, Fujian University of Traditional Chinese Medicine, Fuzhou, Fujian, China; ^2^ Department of Pharmacy, Fuzhou Traditional Chinese Medicine Hospital, Fuzhou, Fujian, China; ^3^ Department of Nephrology, 900 Hospital of the Joint Logistics Team, Fuzhou, China

**Keywords:** isorhamnetin, renal interstitial fibrosis, epithelial mesenchymal transition, TWEAK, Fn14

## Abstract

**Introduction:**

Isorhamnetin (ISO), a prominent active compound found in the fruits of *Hippophae rhamnoides* L., exhibits various pharmacological activities. Recent studies have demonstrated that ISO possesses a significant renoprotective effect. Nevertheless, the specific targets and mechanisms through which ISO exerts its effects against renal interstitial fibrosis (RIF) remain insufficiently explored. The aims of this study were to explore the protective effects of ISO regulating epithelial–mesenchymal transition (EMT) and relieving RIF and to elucidate the underlying molecular mechanisms involved in the tumor necrosis factor-like weak inducer of apoptosis (TWEAK)/fibroblast growth factor-inducible molecule 14 (Fn14) pathway.

**Methods:**

We explored the potential effects and mechanisms of ISO on RIF by using an *in vitro* EMT model of a transforming growth factor-β (TGF-β)-induced human proximal tubular cell line (HK-2) and an *in vivo* unilateral ureteral obstruction (UUO) model. The potential mechanism of the TWEAK/Fn14 pathway involving the protective action of ISO on renal tubules was explored by surface plasmon resonance (SPR) analysis and Fn14 overexpression on UUO rats.

**Results:**

Our findings reveal that ISO can enhance cell morphology and effectively inhibit the migration ability of TGF-β-induced HK-2 cells. ISO also improved renal dysfunction and reduced tubular damage induced by UUO, significantly increasing E-cadherin expression and decreasing α-smooth muscle actin (α-SMA) and the main component of the ECM [type III collagen (Col III) and fibronectin (FN)] *in vivo*. The results show that ISO demonstrates potent inhibition of EMT in renal tubular epithelial cells, both *in vivo* and *in vitro*. The specific interaction between ISO and Fn14 was confirmed by SPR analysis. Overexpression of Fn14 counteracts the renoprotective effects of ISO, mitigating its influence on the inactivation of the TWEAK/Fn14 signaling pathway.

**Conclusions:**

These confirmed that ISO inhibits the EMT of renal tubular epithelial cells by suppressing the TWEAK/Fn14 signaling pathway.

## 1 Introduction

Chronic kidney disease (CKD) has emerged as a public health concern owing to its low cure rate and high rates of morbidity and mortality, with its incidence steadily rising each year (1). Renal interstitial fibrosis (RIF) is the final stage in the progression of CKD to end-stage renal disease, regardless of the initial cause. The assessment of RIF, which reflects the extent of kidney function decline, plays a vital role in guiding therapeutic and prognostic decisions in clinical settings (2). Hence, exploring more effective methods to prevent and address RIF is highly important in the treatment of CKD.

RIF is characterized by excessive myfibroblast (MFB) accumulation, inflammatory cell infiltration, and extracellular matrix (ECM) deposition (3). The renal interstitium is a specialized connective tissue structure in the kidney, primarily composed of sparse reticular and collagen fibers, which provide mechanical support and a microenvironment for renal tubules and vasculature. The excessive deposition of ECM in the renal interstitium drives pathological proliferation and fibrosis, ultimately compressing renal units and impairing kidney function. Therefore, the deposition of ECM is the key factor that induces RIF. MFB is the main effector cell that synthesizes and secretes ECM during the RIF process (4). These MFBs are primarily derived from epithelial–mesenchymal transition (EMT) (5, 6). During EMT, renal tubular epithelial cells undergo a progressive transformation, losing epithelial characteristics such as reduced E-cadherin expression while gaining mesenchymal features, including increased α-smooth muscle actin (α-SMA) expression (7). α-SMA-positive tubular epithelial cells migrate to the renal interstitium, where they transform into myofibroblasts, which then leads to the overproduction of ECM. This disrupts normal tissue structure and contributes to the pathogenesis of RIF (8, 9). Therefore, inhibiting the EMT process may represent a promising therapeutic target for the prevention and treatment of RIF (10).

*Hippophae fructus* (sea buckthorn), the ripe fruit of *Hippophae rhamnoides* L., is widely utilized as a food and health supplement globally, owing to its rich nutritional and medicinal properties (11, 12). The total flavonoids from *H. rhamnoides* L. were reported to have significant renoprotective effects (13, 14). Isorhamnetin (ISO), a significant active flavonoid derived from *H. fructus*, exhibits various pharmacological activities (15). Researchers discovered that ISO demonstrates significant renoprotective effects in ischemia–reperfusion-induced acute kidney injury (16) and in a type 2 diabetic rat model (17). Additionally, ISO exhibits potent antifibrotic activity in both bleomycin-induced pulmonary fibrosis (18). Our previous study demonstrated that ISO suppresses unilateral ureteral obstruction (UUO)-induced renal fibrosis (19), while the specific targets and mechanisms through which ISO exerts its effects against RIF remain insufficiently explored.

Recently, the tumor necrosis factor-like weak inducer of apoptosis (TWEAK)/fibroblast growth factor-inducible molecule 14 (Fn14) pathway has gained increasing importance in RIF (20, 21). However, the precise role of the TWEAK/Fn14 pathway in its modulation of EMT is still unknown. The activation of the TWEAK/Fn14 pathway is regulated by the levels of Fn14 expression (22). The study combined surface plasmon resonance (SPR) measurements of ISO and Fn14 interaction with *in vitro* and *in vivo* models [transforming growth factor-β (TGF-β)-induced human proximal renal tubular epithelial (HK-2) cells and UUO-induced RIF rats] to comprehensively evaluate the target of action and the underlying mechanisms mediating ISO’s modulation of EMT. These data strongly support ISO as a promising therapeutic candidate for RIF.

## 2 Methods

### 2.1 Cells and culture conditions

HK-2 cells (Procell Life Science & Technology Co., Ltd., Wuhan, China) were cultured in minimum essential medium (MEM) containing 10% fetal bovine serum (FBS). Exponentially growing cells were randomly divided into six groups: the control group (untreated), the TGF-β1 group (treated with 10 ng/mL TGF-β1), three ISO pretreatment groups (TGF-β1 + 2, 4, and 8 μmol/L ISO, respectively), and a positive control group (TGF-β1 + 1 μmol/L losartan).

### 2.2 Phalloidin staining of the cell morphology

HK-2 cells were seeded and cultured until the confluence reached 60%. After 48 h, the cells in all treatments were fixed and permeabilized. To analyze the effect of ISO on cell morphology, the cytoskeleton of cells was labeled with 5 mM FITC-phalloidin (Lamboulide Biotechnology Co., Ltd., Beijing, China). The nuclei were stained using 4′,6-diamidino-2-phenylindole (DAPI). Images were captured with a laser confocal microscope (Leica, Wetzlar, Germany).

### 2.3 Cell migration assay

Cells were seeded until their confluence reached nearly 80%. A 10-μL micropipette tip was used to create equal-sized scratches through the cell monolayer. Following 12 h of serum starvation for synchronization, cells were treated with or without TGF-β1 and ISO. Cells were cultured in Dulbecco’s modified Eagle’s medium (DMEM) without FBS and observed under an inverted microscope (Carl Zeiss Microscopy GmbH, Oberkochen, Germany). Images were captured for each group at 0 and 8 h. The width of the scratch was then measured using ImageJ. Cell migration distance = width of the scratch at time 0 − width of the scratch at time 8 h.

### 2.4 Enzyme-linked immunosorbent assay

Upon completion of the cell treatments, the supernatants were collected. The concentrations of type III collagen (Col III) and fibronectin (FN) were measured according to the manufacturer’s instructions.

### 2.5 Animals

SPF-grade male SD rats (180 ± 20 g) were acquired from Shanghai SLAC Laboratory Animal Co. Ltd. [Production License: SCXK (Shanghai) 2019-0002]. All rats were housed in the Experimental Animal Center of Fujian University of Traditional Chinese Medicine [License: SYXK(Min)2019-0007]. All animal care and experimental procedures followed the Institutional Animal Care and Use Committee (IACUC) guidelines and ethical standards and were approved by the Fujian University of Traditional Chinese Medicine (ethics approval number FJTCM IACUC 2022124).

### 2.6 Establishment of the UUO model

SD rats were used to establish the UUO model as reported previously (23). Briefly, the left kidney and ureter were exposed after anesthesia. The left ureter was ligated with 4–0 silk at two points: (a) proximal to the renal pelvis (1 cm from the ureteropelvic junction) and (b) at the mid-ureteric segment (one-third of the ureteral length from the renal pelvis to the bladder). The ureter was then severed between the two ligatures. Then, the ligated kidney was placed gently back into its correct anatomical position, and the incisions were sutured. Sham-operated rats underwent the same procedure, but without ureter ligation.

### 2.7 Adeno-associated virus injections in rats

For overexpression of Fn14 in the kidney, adeno-associated virus 9 (AAV9)-mediated vectors were injected into the renal cortex. AAV2/9-CMV-Rat-T2a-ZsGreen (AAV-NC) and AAV2/9-CMV-Rat-FN14-T2a-ZsGreen (AAV-Fn14) were provided by Hanbio Biotechnology (Shanghai, China) at a titer of 1×10^13^vg/mL. After anesthesia was administered, the left kidney of each rat was exposed, followed by injection with infectious viral particles at five specific locations (10 μL per site), using a 26G 1-mL Sub-Q syringe. The injections, containing either vehicle, AAV2/9-ZsGreen, or AAV2/9-Fn14, were given a 3-week period for expression. Then, the efficacy of virus transfection was confirmed by Western blot before UUO modeling.

### 2.8 Animal groups and administration

#### 2.8.1 Experiment 1

**Figure 1:**
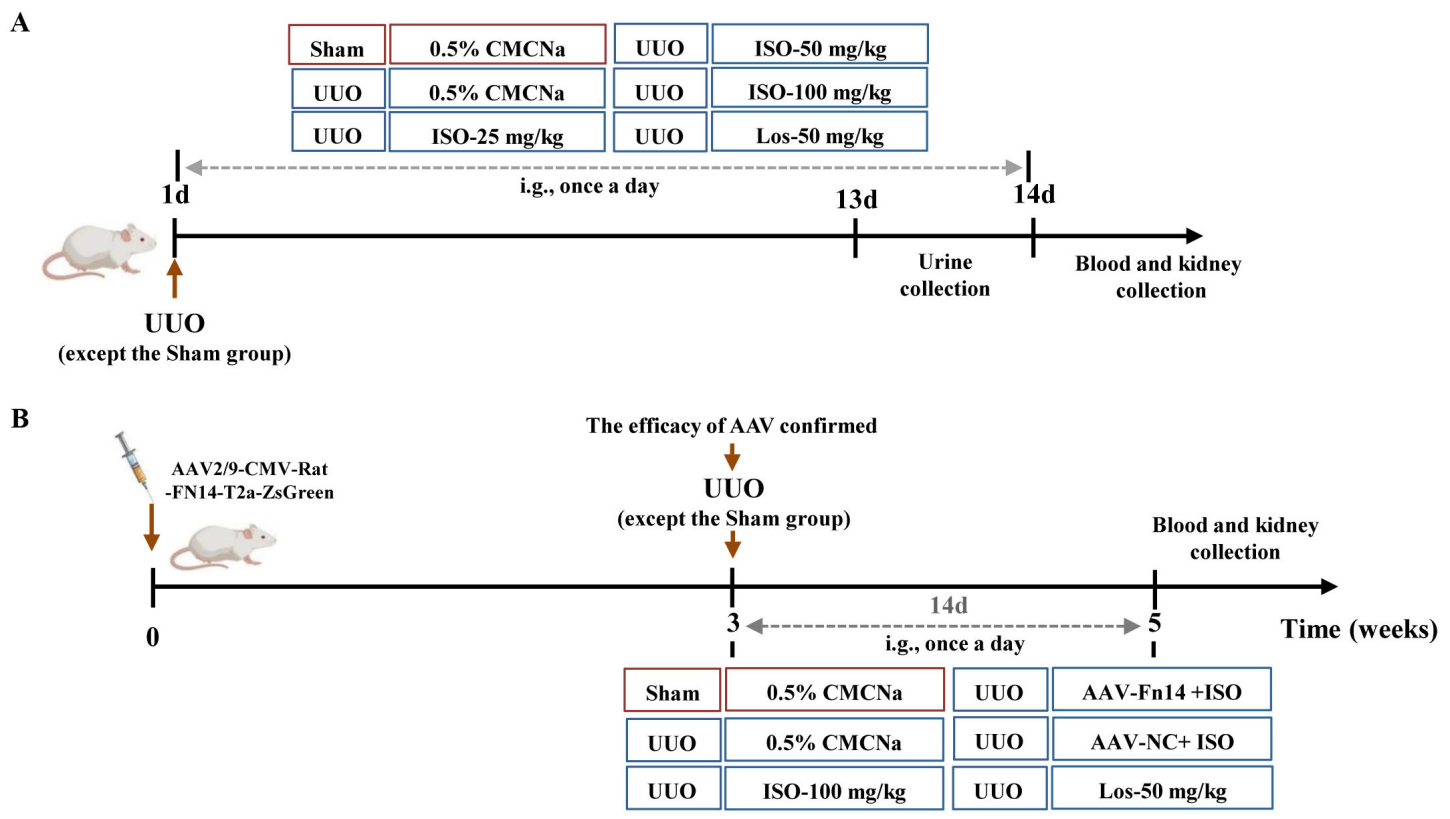
Schedule of the animal experiment. **(A)** The therapeutic effect of ISO (25, 50, and 100 mg/kg) on UUO rats. **(B)** Overexpression of Fn14 dismisses the effectiveness of ISO in UUO rats.

UUO rats were randomly assigned to five groups (*n* = 10): the UUO group, the 25 mg/kg ISO group, the 50 mg/kg ISO group, the 100 mg/kg ISO group, and the 50 mg/kg losartan (Los) group. Ten rats were included in the sham-operated group. Treatment commenced on the day of surgery and was intragastrically administered once daily for a period of 14 days (**Figure 1A**).

#### 2.8.2 Experiment 2

Following AAV injections, a UUO model was established, and the rats were randomly assigned to one of the following groups: the UUO group, the 100 mg/kg ISO group, the AAV-Fn14 + 100 mg/kg ISO group, the AAV-NC + 100 mg/kg ISO group, and the 50 mg/kg Los group. The sham-operated group underwent the same surgical procedures as the UUO group, with the exception of ureter ligation. The treatment was administered according to the same procedure described in Section 2.8.1 (**Figure 1B**).

### 2.9 Assessment of kidney function

After administration on the 13th day, urine was collected for 24 h. The activity of urine N-acetyl-β-D-glucosaminidase (NAG) was determined with colorimetry using available kits (Jiancheng, Nanjing, China). Two hours after the last administration on the 14th day, the rats were anesthetized and blood was collected. Supernatant after centrifugation of blood was used to detect the concentrations of creatinine (SCr) and blood urea nitrogen (BUN) with an assay kit (Jiancheng, Nanjing, China).

The kidneys were rapidly excised after blood collection, weighed, and placed on ice. The kidney index (KI) was calculated as kidney weight/body weight × 100%. The appearance and size of the kidneys, and the thickness of the renal parenchyma of each group of rats were observed.

### 2.10 Hematoxylin and eosin (HE) staining and Masson’s trichrome (Masson) staining

The paraffin sections of kidney tissues were dewaxed, hydrated, and stained with HE and Masson following standard procedures; then, the sections were inspected using a digital camera (Nikon, Tokyo, Japan). Fibrosis was evaluated using the collagen volume fraction (CVF): the percentage of staining with green. The CVF in the left kidney tissue of the rats was evaluated using ImageJ 24.0 software.

### 2.11 Immunohistochemistry analysis

The paraffin sections were dewaxed, hydrated, and subjected to antigen retrieval in a microwave. The primary antibodies used in this study were against Col III (1:1,500; Proteintech, Wuhan, China) and FN (1:400; Proteintech, Wuhan, China), incubated for 1 h. Sections were incubated with secondary antibody for 30 min followed by incubation with DAB and hematoxylin. Images were captured with a Nikon microscope. The percentage of positively stained area was calculated using ImageJ 24.0 software.

### 2.12 Immunofluorescence analysis

The paraffin sections were dewaxed, rehydrated, and then subjected to antigen retrieval. After being blocked with 5% bovine serum albumin (BSA) for 2 h, specific primary antibodies against α-SMA (1:400, Cohesion Biosciences, London, UK) and E-cadherin (1:250, Cohesion Biosciences, London, UK) were incubated, respectively. Sections were covered with the fluorescent secondary antibody. The nuclei were stained with DAPI solution (protected from light). The positive cells were observed under a fluorescence microscope and evaluated using ImageJ 24.0 software.

### 2.13 Western blot analysis

Protein of renal tissues or HK-2 cells was extracted, and detected by the BCA method. After electrophoresis and membrane transfer, the following primary antibodies were used: rabbit polyclonal anti-TNFSF12, anti-SP1, anti-phospho-SP1-T739 (ABclonal, Wuhan, China), anti-TWEAKR (BOSTER Biological Technology Co. Ltd., Wuhan, China), anti-E-cadherin, anti-PCNA, anti-TRAF2 (Cohesion Biosciences, London, UK), anti-ERK1+ERK2, anti-BRAF (Abcam, Danvers, MA), anti-MEK1/2, anti-phospho-ERK1/2 (Thr202/Tyr204), anti-Snail (P, Wuhan, China), anti-phospho-MEK1/2Ab (Affinity Biosciences, Jiangsu, China), mouse anti-α-SMA (Cohesion Biosciences, London, UK), and anti-β-actin (Proteintech, Wuhan, China). The membranes were incubated with the appropriate secondary antibody the following day. Finally, the blots were visualized with an ECL kit (Beyotime Biotechnology, Shanghai, China). The gray value of each band was analyzed.

### 2.14 SPR measurements

SPR measurements were performed with a Biacore T200 system as previously reported (Chen et al., 2023). The Fn14 protein was immobilized on a Chip CM5 (GE Healthcare, Sweden). ISO was injected onto the CM5 chip at various concentrations (0.0625 μM to 0.064 mM). The Biacore T200 evaluation software was used to determine the interaction mode and kinetic constant of ISO with Fn14 (GE Healthcare, Sweden).

### 2.15 Statistical analysis

The data were presented as means ± SD and analyzed using SPSS 26.0 (SPSS Inc., Chicago, IL). Data normality was assessed using the Shapiro–Wilk test. The normally distributed data were compared using analysis of variance (ANOVA). *Post-hoc* analysis was then performed using the LSD method for data with equal variances and the Games–Howell method for data with unequal variances. If data were not normally distributed, the Kruskal–Wallis test was applied. *p*-values below 0.05 indicated significance.

## 3 Results

### 3.1 ISO alleviated morphological changes and improved renal function in the UUO model

**Figure 2:**
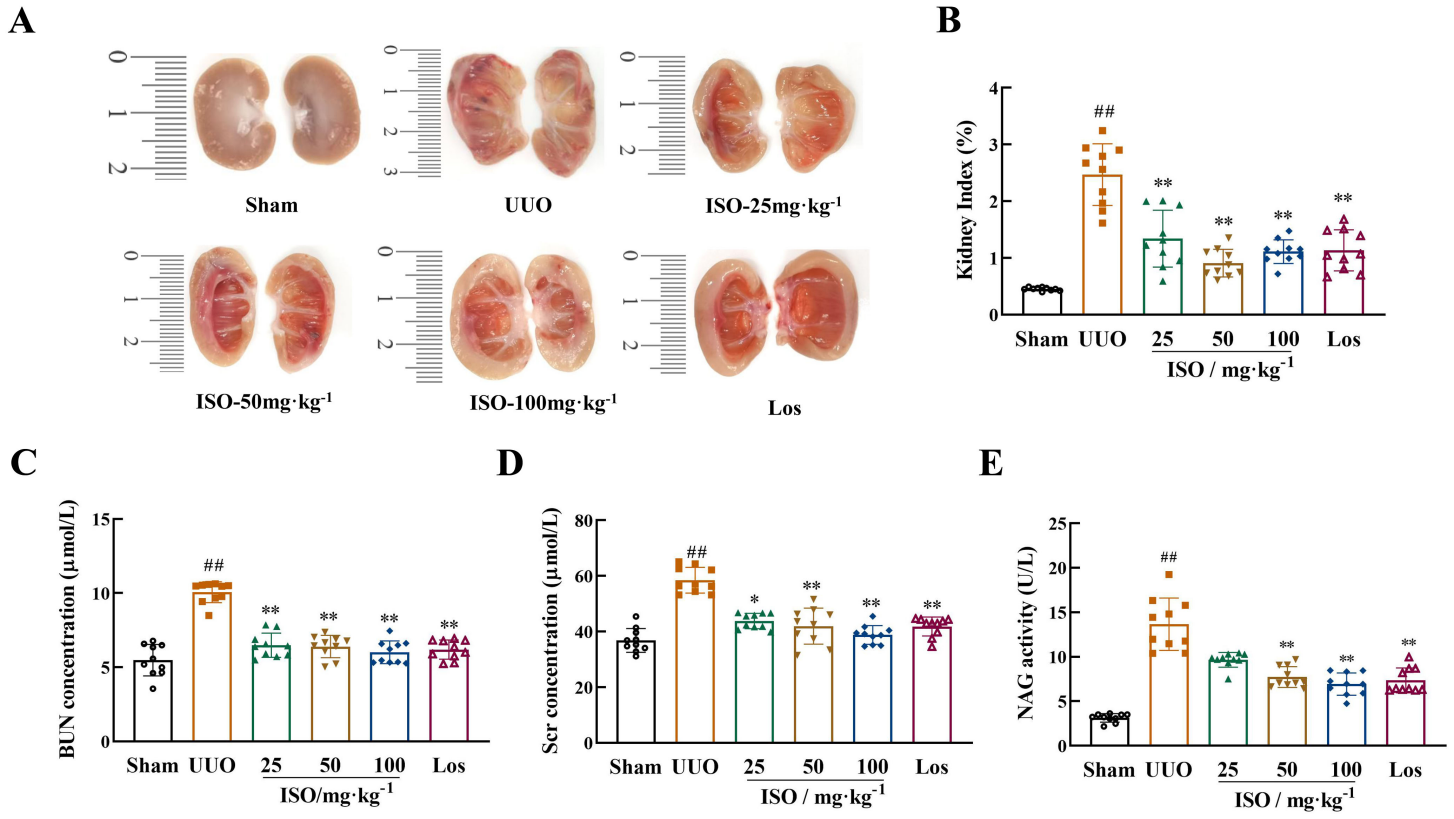
Renoprotective effects of ISO in UUO rats. **(A)** Longitudinal cross-section of the obstructed kidney. **(B)** KI in different groups. **(C, D)** Serum Scr and BUN levels in different groups. **(E)** Urine NAG levels in different groups. Data are expressed as mean ± SD (*n* = 10). ^##^ *p *< 0.01 versus the sham group, ^*^ *p *< 0.05, ^**^ *p *< 0.01 versus the UUO group.

The kidneys in the sham group appeared paired and bean-shaped, had a normal volume, and were covered by a tough capsule (**Figure 2A**). The obstructed kidneys in the UUO group displayed significant enlargement with an uneven surface. The thickened capsule was tightly adhered to the renal parenchyma, and the dissected kidney contained turbid fluid. The cortex and medulla were poorly demarcated, with a thinned cortex, enlarged renal pelvis, and calyces. These changes were mitigated following ISO or Los treatment. **Figure 2B** shows that the UUO group had a significantly higher KI compared to the sham group (*p* < 0.01). Moreover, a statistically significant difference in KI was observed between the treatment groups and the UUO group (*p* < 0.01).

Levels of Scr and BUN in serum and NAG activity (*p* < 0.01) in urine samples exhibited a significant increase in the UUO model group. However, both ISO and Los treatment reduced the levels of Scr and BUN notably, as did the urinary levels of NAG, in model rats (*p* < 0.05 or 0.01) (**Figures 2C–E**).

**Figure 3:**
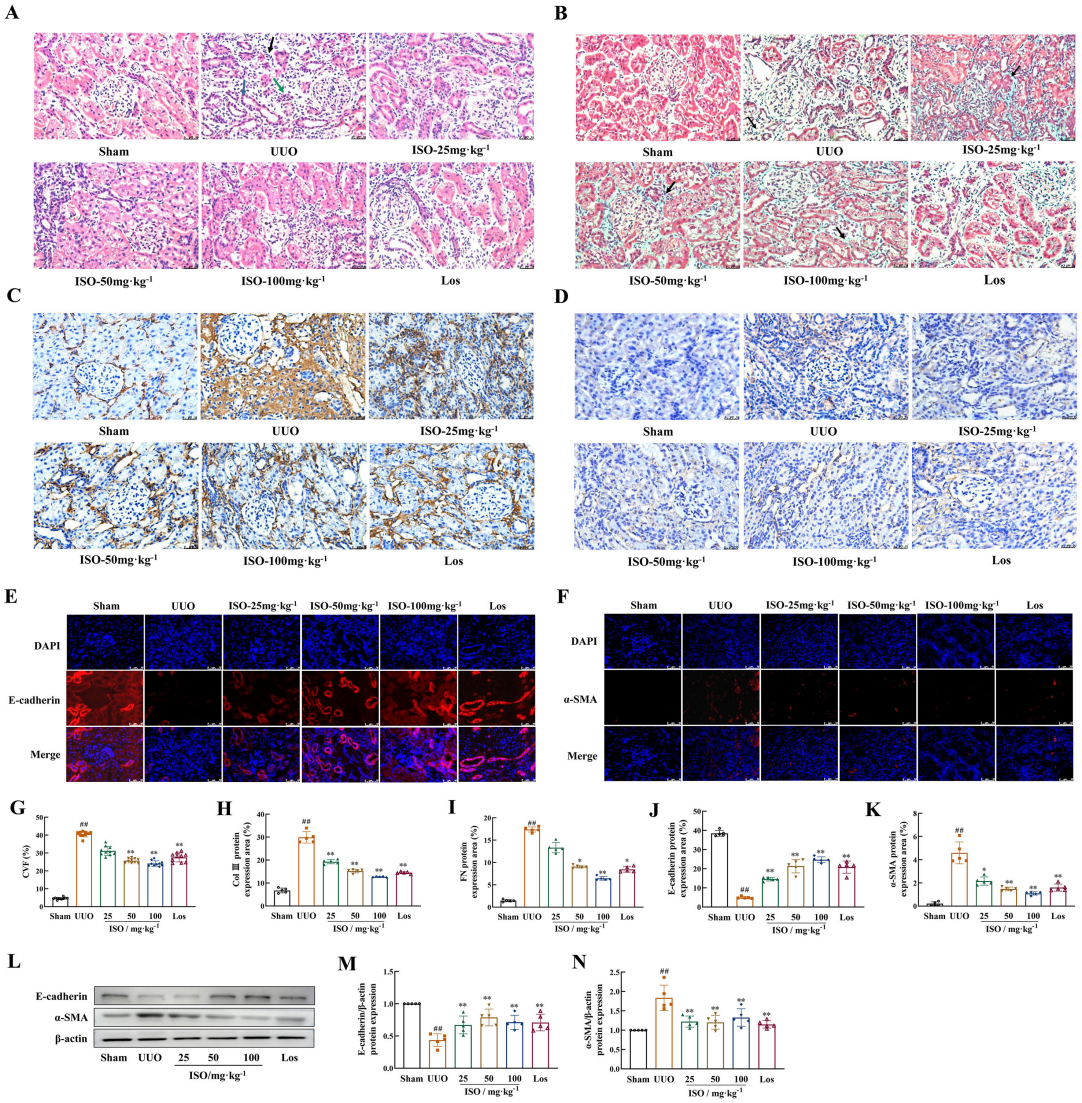
Effects of ISO on renal fibrosis and the development of EMT in UUO rats. **(A)** HE staining of histological changes (×400). Black arrows indicate inflammatory cell infiltrations; the blue arrows indicate dilated tubules, and the green arrows indicate atrophied tubules. **(B, G)** Representative images and the CVF of Masson staining (×400). The green area marked by the black arrows shows collagen fiber deposition (light green dye was used for collagen staining in this experiment). **(A-D)** Representative and quantification analysis of Col III **(C, H)** and FN **(D, I)** immunohistochemical staining (×400). Representative and quantification analysis of E-cadherin **(E, J)** and α-SMA **(F, K)** immunofluorescent staining (×400). Nuclei were counterstained with DAPI (blue). **(L-N)** Western blot and the relative optical densities analysis of E-cadherin and α-SMA in kidney tissue; β-actin was used as the loading control. Data were shown as mean ± SD (*n* = 5). ^##^ *p *< 0.01 versus the sham group, ^*^ *p *< 0.05, ^**^ *p *< 0.01 versus the UUO group.

HE staining was used to assess pathological changes in rat kidney. UUO rats exhibited significant renal structural damage. This damage was characterized by cystic dilation of renal tubular lumens, tubular atrophy, and occasional protein casts. Additionally, there was increased inflammatory cell infiltration and fibrous tissue hyperplasia in renal interstitium. These pathological changes were improved after ISO or Los treatment (**Figure 3A**). Furthermore, Masson staining revealed abundant green-stained collagen fibers deposited in the renal interstitium, and CVF increased significantly in the UUO rats (*p* < 0.01). Following ISO or Los treatment, fewer collagen fiber streaks (green-stained areas) were observed and CVF was decreased (*p* < 0.01) (**Figures 3B, G**). These findings suggest that both ISO and Los ameliorated renal damage and fibrosis in UUO rats.

### 3.2 ISO inhibited ECM deposition and suppressed EMT in UUO rats

The effects of ISO on the generation of ECM and the occurrence and development of EMT in UUO rats were observed. Immunohistochemistry revealed that Col III and FN were markedly upregulated in UUO kidneys while ISO or Los treatment effectively restored their expression (*p* < 0.05 or 0.01) (**Figures 3C, D, H, I**). These results collectively demonstrated that both ISO and Los can significantly reduce UUO-induced accumulation of ECM. Both immunofluorescence staining (**Figures 3E, F, J, K**) and Western blot detection (**Figures 3L–N**) indicated a visible decrease in E-cadherin and a visible increase in the accumulation of α-SMA in the UUO group (*p* < 0.01). Expression of E-cadherin and α-SMA was significantly mitigated in the treated groups of UUO rats (*p* < 0.05 or 0.01).

### 3.3 ISO inhibited ECM deposition and suppressed EMT in HK-2 cells

**Figure 4:**
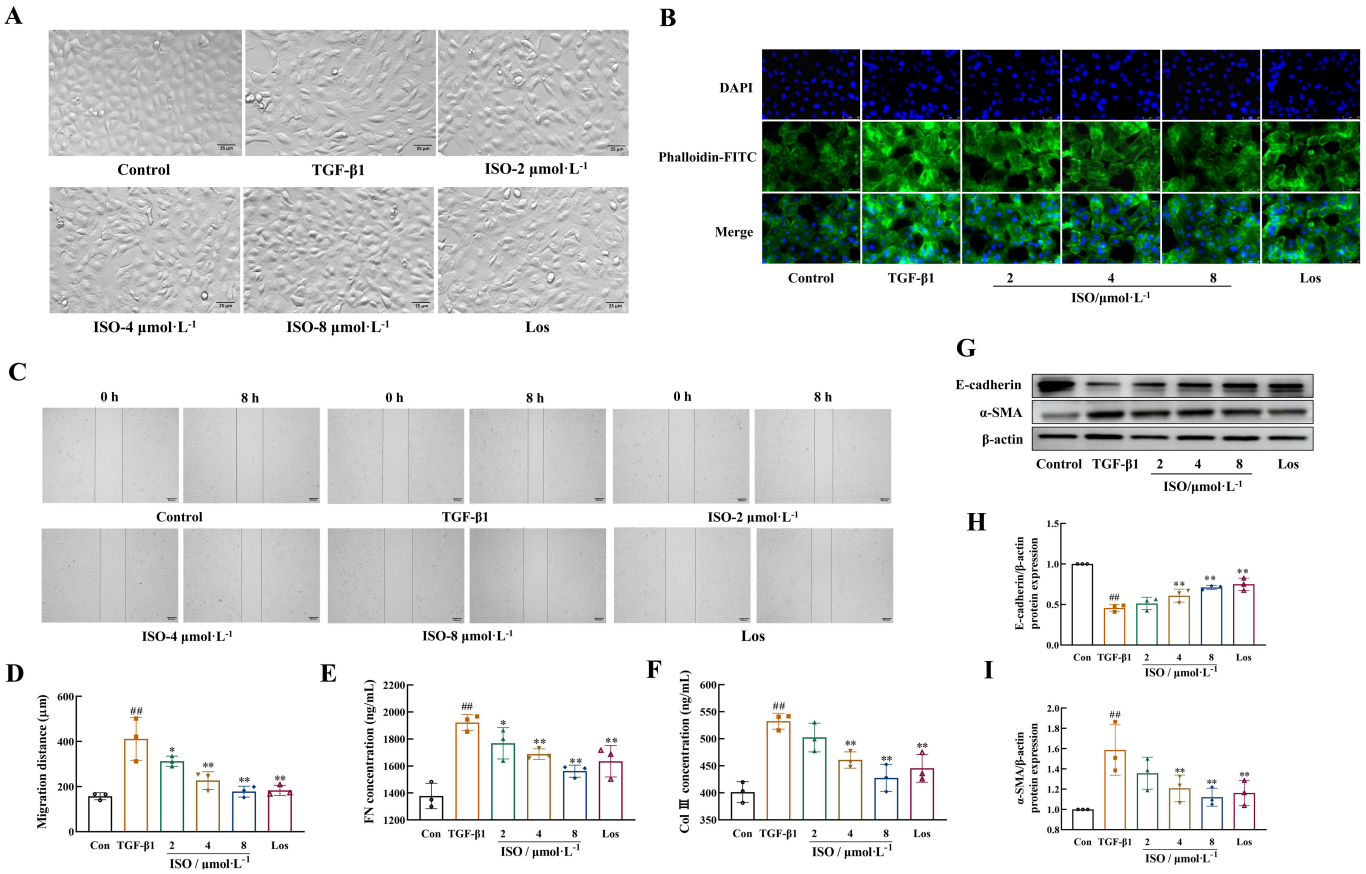
ISO attenuated TGF-β1-induced EMT in HK-2 cells. **(A)** The cellular morphology of HK-2 cells (×200). **(B)** Representative images showing the cytoskeleton of HK-2 cells (×400). The cytoskeletons were stained with phalloidin (green) and nuclei were stained with DAPI (blue). **(C, D)** The cell migration ability was evaluated by the scratch assay (×50). **(E, F)** Levels of Col III and FN in the cell supernatant were detected by enzyme-linked immunosorbent assay (ELISA). **(G–I)** Western blot and the relative optical densities analysis of E-cadherin and α-SMA; β-actin was used as the loading control. Data were shown as mean ± SD (*n* = 3). ^##^ *p *< 0.01 versus the sham group, ^*^ *p *< 0.05, ^**^ *p *< 0.01 versus the UUO group.

Results of the HK-2 cells in the control group presented a cobblestone morphology characteristic of epithelial cells. The cells in the TGF-β1 group exhibited an elongated, spindle-like morphology resembling fibroblasts, with widened intercellular spaces, and these changes were mitigated by treatment with ISO or Los (**Figure 4A**).

Phalloidin was used to specifically label the cytoskeleton. HK-2 cells in the control group exhibited a clear and complete cytoskeleton structure, arranged in an orderly and uniform manner. After 48-h induction with 10 ng/mL TGF-β1, the microfilament cytoskeleton demonstrated disordered organization, characterized by numerous bundled fibrous structures in the cytoplasm. These structures formed a loose network connecting the cell membrane and nucleus, with partial longitudinal alignment along the cellular axis. Treatment with ISO or Los improved the morphological organization of the microfilament skeleton (**Figure 4B**).

After 8 h, the migration distance of HK-2 cells treated with TGF-β1 was markedly greater than that of control HK-2 cells (*p* < 0.01), while the migration distances in the Los group and different doses of ISO groups were significantly lower (*p* < 0.05 or 0.01) (**Figures 4C, D**).

The secretion of extracellular components FN and Col III in the cell supernatant of TGF-β1-induced HK-2 cells was significantly decreased in the ISO group and Los group (*p* < 0.05 or 0.01) (**Figures 4E, F**).

Moreover, after ISO treatment, the protein expression of E-cadherin was significantly increased, and the expression of the mesenchymal phenotype marker protein α-SMA in the cells was obviously decreased compared with the TGF-β1 group (*p* < 0.01) (**Figures 4G–I**).

### 3.4 ISO attenuates the TWEAK/Fn14 pathway *in vitro*

**Figure 5:**
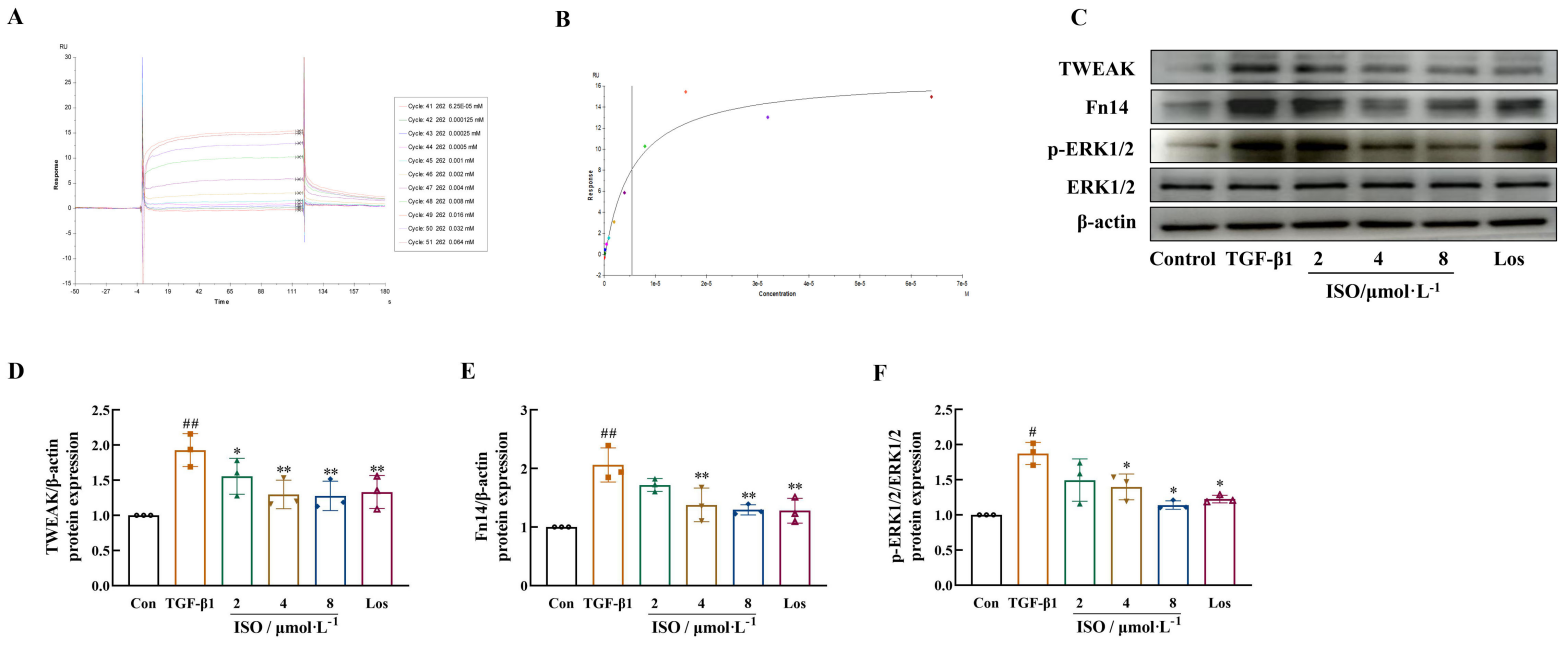
Effects of ISO on the TWEAK/Fn14 signaling pathway in TGF-β1-induced HK-2 cells. **(A, B)** ISO directly interacts with Fn14, dose–response sensorgrams, and the affinity constant (*K*_d_ = 5.47 μM) of ISO with Fn14. **(C–F)** Western blot analysis of TWEAK, Fn14, and the phosphorylation levels of ERK1/2 in TGF-β1-induced HK-2 cells. β-actin was used as the loading control. Data are expressed as mean ± SD (*n* = 3). ^#^ *p *< 0.05, ^##^ *p *< 0.01 versus the sham group, ^*^ *p *< 0.05, ^**^ *p *< 0.01 versus the UUO group.

Results demonstrated the specific binding activity of ISO to Fn14 in a concentration-dependent manner. The maximum response was 14 AU (**Figure 5A**), and the dissociation constant (*K*_d_) was determined to be 5.47 μM (**Figure 5B**).

The effects of ISO on the TWEAK/Fn14/ERK1/2 signaling pathway were observed in HK-2 cells, and the results suggested that ISO treatment inhibited the expression of TWEAK and Fn14 and the phosphorylation of ERK1/2 in TGF-β1-treated HK-2 cells (*p* < 0.05 or 0.01) (**Figures 5C–F**).

### 3.5 ISO inhibits the TWEAK/Fn14 pathway in UUO rats

**Figure 6:**
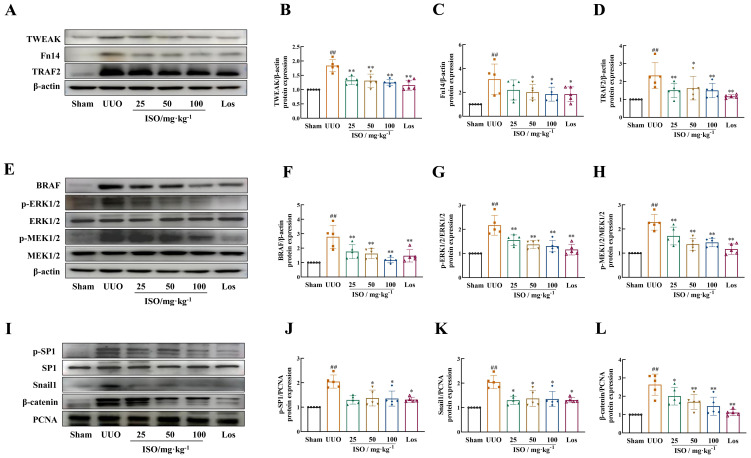
Effect of ISO on the expression of TWEAK/Fn14 signaling pathway-related proteins in UUO rats. Representative images and quantification of Western blot results of the protein expression of TWEAK, Fn14, TRAF2, and BRAF **(A–D)**, and phosphorylation of MER1/2 and ERK1/2 **(E–H)**. β-actin was used as the loading control for total protein. **(I–L)** Relative optical density analysis of the nucleoprotein expression of p-SP1, Snail, and β-catenin. PCNA was used as the loading control for nucleoprotein. All data are expressed as the mean ± SD (*n* = 5). ^##^ *p *< 0.01 versus the sham group, ^*^ *p *< 0.05, ^**^ *p *< 0.01 versus the UUO group.

Further examination of the TWEAK/Fn14 signaling pathways targeted by the ISO pathway was performed *in vivo*. UUO kidneys presented increased expression of TWEAK and Fn14, its downstream signals TNFR-associated factor 2 (TRAF2) and BRAF, and phosphorylated levels of MER1/2 and ERK1/2 (*p* < 0.01). ISO treatment significantly inhibited TWEAK, Fn14, TRAF2, and BRAF expression and suppressed MER1/2 and ERK1/2 phosphorylation (*p* < 0.05 or 0.01) (**Figures 6A–H**). ISO treatment significantly suppressed the expression of Snail1 and β-catenin in the cell nucleus and inhibited the phosphorylation level of SP1 in UUO rats (*p* < 0.05 or 0.01) (**Figures 6I–L**).

### 3.6 Fn14 overexpression compromises the therapeutic effects and regulation of the TWEAK/Fn14 pathway-related proteins of ISO on RIF

**Figure 7:**
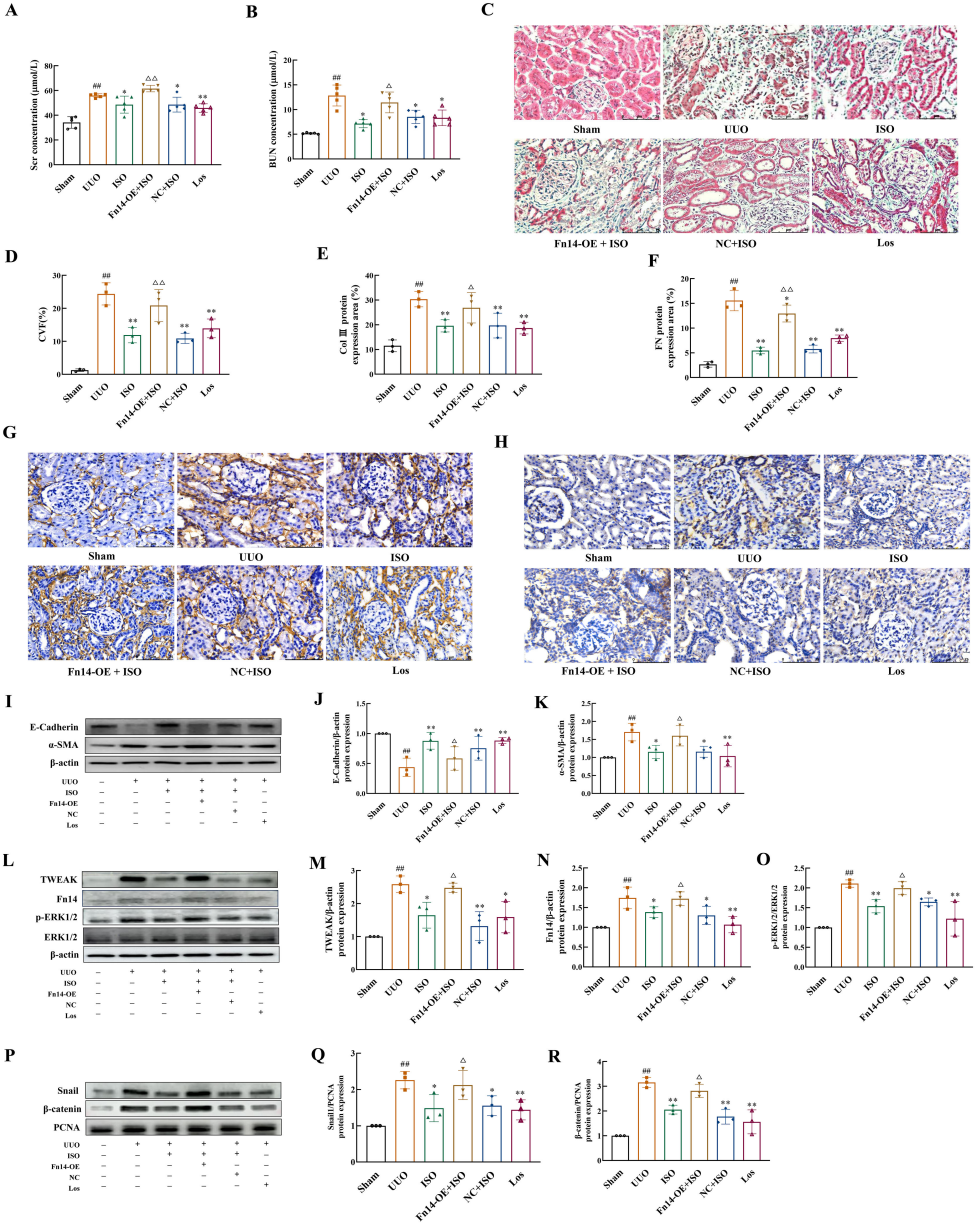
Fn14 overexpression compromises the therapeutic effects of ISO and the regulation of ISO on the TWEAK/Fn14 pathway-related proteins on UUO model rats. **(A, B)** Serum Scr and BUN levels in different groups. **(C, D)** Representative images and the CVF of Masson staining (×400). **(E–H)** Representative and quantification analysis of Col III and FN immunohistochemical staining (×400). Nuclei were counterstained with DAPI (blue). **(I–K)** Western blot and the relative optical densities analysis of E-cadherin and α-SMA in kidney tissue. **(L–O)** Representative images and quantification of Western blot results of the protein expression of TWEAK, Fn14, and phosphorylation of ERK1/2. β-actin was used as the loading control. **(P–R)** Relative optical density analysis of the nucleoprotein expression of Snail and β-catenin. PCNA was used as the loading control for nucleoprotein. Data were shown as mean ± SD (*n* = 3). ^##^ *p *< 0.01 versus the sham group, ^*^ *p *< 0.05, ^**^ *p *< 0.01 versus the UUO group, ^△^ *p *< 0.05, ^△△^ *p *< 0.01 versus the ISO group.

ISO treatment significantly reduced the levels of Scr, BUN, and CVF of UUO rats (*p* < 0.05 or 0.01). Interestingly, Fn14 overexpression completely reversed these beneficial effects of ISO (*p* < 0.05 or 0.01) (**Figures 7A–D**).

ISO treatment inhibited the protein expression of Col III, FN, and α-SMA in the kidney tissue of UUO rats, while increasing the expression of E-cadherin (*p* < 0.05 or 0.01). However, Fn14 overexpression abolished the inhibitory effects of ISO on EMT (*p* < 0.05 or 0.01) (**Figures 7E–K**).

ISO treatment significantly inhibited the protein expression level of TWEAK and Fn14, as well as the phosphorylation level of ERK1/2, and suppressed the nuclear protein expression of Snail1 and β-catenin (*p* < 0.05 or 0.01). However, Fn14 overexpression abrogated the inhibitory effects of ISO on the TWEAK/Fn14 pathway (*p* < 0.05) (**Figures 7L–R**).

## 4 Discussion

Ureteral obstruction disrupts renal homeostasis, leading to impaired urine excretion, hydronephrosis, and the accumulation of filtered metabolites (24). When obstruction persists, the pressure in the renal pelvis continues to rise, which compresses the renal parenchyma, leading to gradual atrophy, thinning, and the infiltration of inflammatory cells into the surrounding interstitial tissue (25). Renal tubular epithelial cells undergo continuous damage, leading to a phenotypic shift that enables them to evade apoptosis through EMT (26). This process promotes the proliferation of myofibroblasts and the accumulation of ECM, ultimately resulting in RIF (27). The UUO model closely resembles histopathological changes to clinical renal tubular injury and interstitial fibrosis, and is frequently used to evaluate the therapeutic potential of novel treatments (28).

This study showed that the left kidney of the UUO group exhibited cystic swelling and significant thinning of renal parenchyma. The observed microscopic features, including tubular atrophy, luminal dilation, interstitial inflammation, and collagen deposition, are consistent with previously reported pathological characteristics of RIF (29). The KI values of the rats in the UUO group, along with their serum Scr and BUN levels, were significantly elevated, indicating that the rats suffered severe renal damage after UUO. Moreover, NAG, a specific and highly sensitive marker of renal tubular damage, is stable in urine, allowing for the reliable detection and monitoring of renal tubular lesion progression. The elevated NAG levels in the UUO group suggested that renal function impairment in the model group primarily affected the renal tubules. ISO treatment provided effective renal tubular protection in UUO rats, as indicated by decreased serum Scr and BUN levels, urinary NAG activity, and the KI value in the model rats. Additionally, ISO significantly alleviated pathological damage in renal tissue on the obstructed side, reduced collagen deposition, and delayed the progression of RIF progression.

As an angiotensin II receptor blocker, Los is commonly used for the treatment of CKD, and its antifibrotic effects on the kidneys are supported by extensive research (30). Los served as a positive control in our experiments to verify the reliability of the experimental system. The analysis revealed no statistically significant difference in efficacy between ISO and Los, suggesting that the therapeutic effects of ISO are similar to those of Los.

EMT is considered the primary pathological mechanism driving ECM deposition in the advancement of RIF. α-SMA and E-cadherin expression changes are well-established molecular markers for EMT identification. Immunofluorescence and Western blot results revealed that the expression of E-cadherin was significantly reduced in rat tubular epithelial cells of the UUO model, while α-SMA and the ECM components (Col III and FN) were significantly increased (*p* < 0.01). These results provided evidence that EMT had occurred within the UUO model. ISO inhibited the expression of α-SMA, Col III, and FN and the absence of E-cadherin, suggesting that its renoprotective effects on RIF involve blocking the phenotypic shift of renal tubular epithelial cells and impeding the progression of EMT.

Our *in vitro* results showed that TGF-β1 stimulation led to marked changes in renal tubular epithelial cells, including a transition to a fibroblast-like morphology, enhanced cell migration, and reduced expression of E-cadherin. In addition, α-SMA and ECM proteins (Col III and FN) exhibited significantly increased expression. ISO treatment slowed the TGF-β1-induced fibroblast-like morphological change and decreased the migration distance of HK-2 cells. Moreover, ISO treatment effectively attenuated TGF-β1-induced downregulation of E-cadherin and upregulation of α-SMA, Col III, and FN, indicating its potential to inhibit EMT progression.

TWEAK, a TNF superfamily cytokine, uniquely interacts with Fn14, its only known transmembrane receptor. This interaction recruits specific binding motifs for TRAF2. Subsequently, TRAF2, acting as an E3 ubiquitin ligase, utilizes its RING finger domain to bind BRAF, promoting BRAF ubiquitination and the downstream phosphorylation of MEK1/2 and ERK1/2. Finally, phosphorylated ERK1/2 translocates to the nucleus, enhancing SP1 activity and further modulating Snail1 expression (31). Notably, Snail1 contributes to the suppression of E-cadherin, resulting in disrupted cell adhesion processes and subsequent loss of epithelial characteristics (32). The decreased intercellular adhesion led to the dissociation of β-catenin and E-cadherin. β-catenin subsequently accumulates in the cytoplasm and translocates into the nucleus, initiating the expression of EMT-related genes including Snail1 and α-SMA (33–35).

SPR was used to investigate the interaction between ISO and Fn14. The results demonstrated that ISO exhibited specific binding activity towards Fn14. In our study, we utilized a TGF-β1-induced HK-2 cell model to examine the effects of ISO on the expression of key factors in the TWEAK/Fn14 pathway. Results indicated that ISO significantly downregulated the protein expression of TWEAK and Fn14, as well as the phosphorylation of ERK1/2.

**Figure 8:**
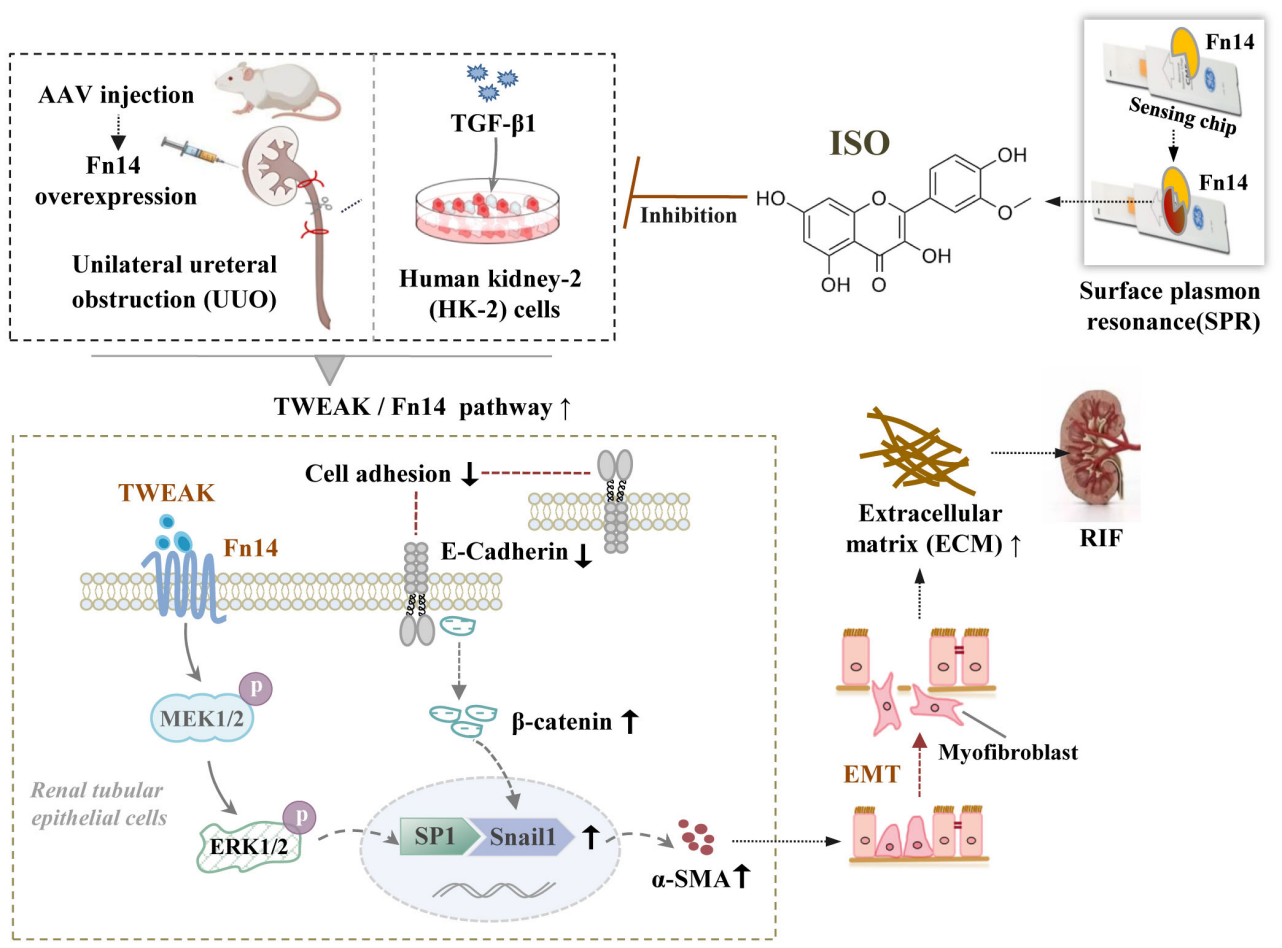
The underlying mechanisms by which isorhamnetin attenuates renal interstitial fibrosis. Schematic diagram illustrating the involvement of the TWEAK/Fn14/ERK1/2 signaling pathway in the renoprotective effects of ISO against RIF by its inhibition on EMT of renal tubular epithelial cells.

To further investigate the mechanism of action and targets of ISO, we conducted animal experiments to detect the related proteins involved in this pathway. ISO significantly downregulated TWEAK and Fn14 protein expression and ERK1/2 protein phosphorylation in the kidney tissue of UUO rats. In addition, ISO significantly downregulated TRAF2 and BRAF protein expression and MEK1/2 protein phosphorylation levels in the kidney tissue of UUO rats while significantly reducing β-catenin and Snail1 protein and SP1 phosphorylation levels in the nucleus. When Fn14 overexpressed, the renoprotective effects of ISO against RIF, specifically its ability to protect renal tubules by inhibiting ECM production and EMT progression, can be reversed. Additionally, the inhibitory effect of ISO on the activation of the TWEAK/Fn14 pathway is also counteracted. The above results illustrate that the observed effects of ISO on EMT are attributed to its ability to inhibit the activation of the TWEAK/Fn14 signaling pathway (**Figure 8**).

## 5 Conclusion

In summary, our results confirm that ISO inhibits the development of EMT and the deposition of ECM both *in vivo* and *in vitro.* Therefore, ISO may be able to protect renal tubules and impede the occurrence and progression of RIF through the TWEAK/Fn14 signaling pathway, providing an experimental basis for clinical applications.

## Data Availability

The original contributions presented in the study are included in the article/supplementary material. Further inquiries can be directed to the corresponding authors.

## References

[1] Diseases GBD Injuries C. Global burden of 369 diseases and injuries in 204 countries and territories, 1990-2019: a systematic analysis for the Global Burden of Disease Study 2019. Lancet. (2020) 396:1204–22. doi: 10.1016/S0140-6736(20)30925-9, PMID: 33069326 PMC7567026

[2] Papasotiriou M Genovese F Klinkhammer BM Kunter U Nielsen SH Karsdal MA. Serum and urine markers of collagen degradation reflect renal fibrosis in experimental kidney diseases. Nephrol Dial Transplant. (2015) 30:1112–21. doi: 10.1093/ndt/gfv063, PMID: 25784725

[3] Humphreys BD. Mechanisms of renal fibrosis. Annu Rev Physiol. (2018) 80:309–26. doi: 10.1146/annurev-physiol-022516-034227, PMID: 29068765

[4] Djudjaj S Boor P. Cellular and molecular mechanisms of kidney fibrosis. Mol Aspects. Med. (2019) 65:16–36. doi: 10.1016/j.mam.2018.06.002, PMID: 29909119

[5] Grande MT Lopez-Novoa JM. Fibroblast activation and myofibroblast generation in obstructive nephropathy. Nat Rev Nephrol. (2009) 5:319–28. doi: 10.1038/nrneph.2009.74, PMID: 19474827

[6] Kim TW Kim YJ Seo CS Kim HT Park SR Lee MY. Elsholtzia ciliata (Thunb.) Hylander attenuates renal inflammation and interstitial fibrosis via regulation of TGF-ss and Smad3 expression on unilateral ureteral obstruction rat model. Phytomedicine. (2016) 23:331–9. doi: 10.1016/j.phymed.2016.01.013, PMID: 27002403

[7] Xue M Cheng Y Han F Chang Y Yang Y Li X. Triptolide attenuates renal tubular epithelial-mesenchymal transition via the miR-188-5p-mediated PI3K/AKT pathway in diabetic kidney disease. Int J Biol Sci. (2018) 14:1545–57. doi: 10.7150/ijbs.24032, PMID: 30263007 PMC6158722

[8] Shibata-Seki T Nagaoka M Goto M Kobatake E Akaike T. Direct visualization of the extracellular binding structure of E-cadherins in liquid. Sci Rep. (2020) 10:17044. doi: 10.1038/s41598-020-72517-2, PMID: 33046720 PMC7552386

[9] Yuan Q Tan RJ Liu Y. Myofibroblast in kidney fibrosis: origin, activation, and regulation. Adv Exp Med Biol. (2019) 1165:253–83. doi: 10.1007/978-981-13-8871-2_12, PMID: 31399969

[10] Allison SJ. Fibrosis: Targeting EMT to reverse renal fibrosis. Nat Rev Nephrol. (2015) 11:565. doi: 10.1038/nrneph.2015.133, PMID: 26241016

[11] Wang Z Zhao F Wei P Chai X Hou G Meng Q. Phytochemistry, health benefits, and food applications of sea buckthorn (Hippophae rhamnoides L.): A comprehensive review. Front Nutr. (2022) 9:1036295. doi: 10.3389/fnut.2022.1036295, PMID: 36562043 PMC9763470

[12] Zhao S Sun H Liu Q Shen Y Jiang Y Li Y. Protective effect of seabuckthorn berry juice against acrylamide-induced oxidative damage in rats. J Food Sci. (2020) 85:2245–54. doi: 10.1111/1750-3841.15313, PMID: 32579735

[13] Lei T Xu D Lei Y Wang JJ Bai Y Ma ZQ. Therapeutic effects and mechanism of total flavonoids from Hippophae Fructus on early diabetic nephropathy in rats. Inf TCM. (2025) 42:36–41. Available online at: https://www.cnki.com.cn/Article/CJFDTotal-ZYXN202507006.htm.

[14] Li YW Tang ZS Zhang Z Liang T Song ZX Wang CL. Effect of total flavonoids of hippophae on the renal fibrosis of unilateral ureteral obstructive and related mechanism. Cent South Pharmacy. (2021) 19:845–50. doi: 10.7539/j.issn.1672-2981.2021.05.010

[15] Gong G Guan YY Zhang ZL Rahman K Wang SJ Zhou S. Isorhamnetin: A review of pharmacological effects. BioMed Pharmacother. (2020) 128:110301. doi: 10.1016/j.biopha.2020.110301, PMID: 32502837

[16] Shujuan Q Yunxia Z Xiangling L Zhentao G MIn G. The immune protective effects of isorhamnetin on ischemia reperfusion induced acute kidney injury in rats. Curr Immunol. (2017) 37:461–6. Available online at: https://en.cnki.com.cn/Article_en/CJFDTotal-SHMY201706004.htm

[17] Qiu S Sun G Zhang Y Li X Wang R. Involvement of the NF-kappaB signaling pathway in the renoprotective effects of isorhamnetin in a type 2 diabetic rat model. BioMed Rep. (2016) 4:628–34. doi: 10.3892/br.2016.636, PMID: 27123259 PMC4840710

[18] Zheng Q Tong M Ou B Liu C Hu C Yang Y. Isorhamnetin protects against bleomycin-induced pulmonary fibrosis by inhibiting endoplasmic reticulum stress and epithelial-mesenchymal transition. Int J Mol Med. (2019) 43:117–26. doi: 10.3892/ijmm.2018.3965, PMID: 30387812 PMC6257865

[19] Guan HX Nan LH Chen YP Zhang YQ Lou XH Cui T. Mechanism study of isorhamnetin inhibiting renal interstitial fibrosis in UUO rats. Fujian. J TCM February. (2023) 54:43–7. doi: 10.13260/j.cnki.jfjtcm.2023.02011

[20] Berzal S Gonzalez-Guerrero C Rayego-Mateos S Ucero A Ocana-Salceda C Egido J. TNF-related weak inducer of apoptosis (TWEAK) regulates junctional proteins in tubular epithelial cells via canonical NF-kappaB pathway and ERK activation. J Cell Physiol. (2015) 230:1580–93. doi: 10.1002/jcp.24905, PMID: 25536182

[21] Di Martino L Osme A Kossak-Gupta S Pizarro TT Cominelli F. TWEAK/fn14 is overexpressed in crohn’s disease and mediates experimental ileitis by regulating critical innate and adaptive immune pathways. Cell Mol Gastroenterol Hepatol. (2019) 8:427–46. doi: 10.1016/j.jcmgh.2019.05.009, PMID: 31181286 PMC6718944

[22] Dohi T Burkly LC. The TWEAK/Fn14 pathway as an aggravating and perpetuating factor in inflammatory diseases: focus on inflammatory bowel diseases. J Leukoc Biol. (2012) 92:265–79. doi: 10.1189/jlb.0112042, PMID: 22672874

[23] Yuan J Shen Y Yang X Xie Y Lin X Zeng W. Thymosin beta4 alleviates renal fibrosis and tubular cell apoptosis through TGF-beta pathway inhibition in UUO rat models. BMC Nephrol. (2017) 18:314. doi: 10.1186/s12882-017-0708-1, PMID: 29047363 PMC5648500

[24] Norregaard R Mutsaers HAM Frokiaer J Kwon TH. Obstructive nephropathy and molecular pathophysiology of renal interstitial fibrosis. Physiol Rev. (2023) 103:2827–72. doi: 10.1152/physrev.00027.2022, PMID: 37440209 PMC10642920

[25] Chevalier RL Forbes MS Thornhill BA. Ureteral obstruction as a model of renal interstitial fibrosis and obstructive nephropathy. Kidney Int. (2009) 75:1145–52. doi: 10.1038/ki.2009.86, PMID: 19340094

[26] Liu BC Tang TT Lv LL Lan HY. Renal tubule injury: a driving force toward chronic kidney disease. Kidney Int. (2018) 93:568–79. doi: 10.1016/j.kint.2017.09.033, PMID: 29361307

[27] Hammad FT. The long-term renal effects of short periods of unilateral ureteral obstruction. Int J Physiol Pathophysiol. Pharmacol. (2022) 14:60–72. Available online at: https://pubmed.ncbi.nlm.nih.gov/35619661/. 35619661 PMC9123473

[28] Mao Y Yu J Da J Yu F Zha Y. Acteoside alleviates UUO-induced inflammation and fibrosis by regulating the HMGN1/TLR4/TREM1 signaling pathway. PeerJ. (2023) 11:e14765. doi: 10.7717/peerj.14765, PMID: 36691481 PMC9864189

[29] Li T Yang K Tong Y Guo S Gao W Zou X. Targeted drug therapy for senescent cells alleviates unilateral ureteral obstruction-induced renal injury in rats. Pharmaceutics. (2024) 16:695. doi: 10.3390/pharmaceutics16060695, PMID: 38931822 PMC11206309

[30] Zou J Zhou X Ma Y Yu R. Losartan ameliorates renal interstitial fibrosis through metabolic pathway and Smurfs-TGF-beta/Smad. BioMed Pharmacother. (2022) 149:112931. doi: 10.1016/j.biopha.2022.112931, PMID: 36068784

[31] Qian Y Yao W Yang T Yang Y Liu Y Shen Q. aPKC-iota/P-Sp1/Snail signaling induces epithelial-mesenchymal transition and immunosuppression in cholangiocarcinoma. Hepatology. (2017) 66:1165–82. doi: 10.1002/hep.29296, PMID: 28574228

[32] Park MJ Lee DE Shim MK Jang EH Lee JK Jeong SY. Piperlongumine inhibits TGF-beta-induced epithelial-to-mesenchymal transition by modulating the expression of E-cadherin, Snail1, and Twist1. Eur J Pharmacol. (2017) 812:243–9. doi: 10.1016/j.ejphar.2017.07.036, PMID: 28734931

[33] Li Z Zhou L Wang Y Miao J Hong X Hou FF. (Pro)renin receptor is an amplifier of wnt/beta-catenin signaling in kidney injury and fibrosis. J Am Soc Nephrol. (2017) 28:2393–408. doi: 10.1681/ASN.2016070811, PMID: 28270411 PMC5533230

[34] Tan RJ Zhou D Zhou L Liu Y. Wnt/beta-catenin signaling and kidney fibrosis. Kidney Int Suppl. (2014) 4:84–90. doi: 10.1038/kisup.2014.16, PMID: 26312156 PMC4536962

[35] Wu WS You RI Cheng CC Lee MC Lin TY Hu CT. Snail collaborates with EGR-1 and SP-1 to directly activate transcription of MMP 9 and ZEB1. Sci Rep. (2017) 7:17753. doi: 10.1038/s41598-017-18101-7, PMID: 29259250 PMC5736704

